# Method-Dependent Implications in Foodborne Pathogen Quantification: The Case of *Campylobacter coli* Survival on Meat as Comparatively Assessed by Colony Count and Viability PCR

**DOI:** 10.3389/fmicb.2021.604933

**Published:** 2021-03-01

**Authors:** Thomai P. Lazou, Athanasios I. Gelasakis, Serafeim C. Chaintoutis, Eleni G. Iossifidou, Chrysostomos I. Dovas

**Affiliations:** ^1^Laboratory of Hygiene of Foods of Animal Origin – Veterinary Public Health, Faculty of Health Sciences, School of Veterinary Medicine, Aristotle University of Thessaloniki, Thessaloniki, Greece; ^2^Laboratory of Anatomy and Physiology of Farm Animals, Department of Animal Science, School of Animal Biosciences, Agricultural University of Athens, Athens, Greece; ^3^Diagnostic Laboratory, Faculty of Health Sciences, School of Veterinary Medicine, Aristotle University of Thessaloniki, Thessaloniki, Greece

**Keywords:** *Campylobacter*, meat, viability PCR, propidium monoazide, viable but non-culturable

## Abstract

The aim of the present study was to address method-dependent implications during the quantification of viable *Campylobacter coli* cells on meat over time. Traditional colony counting on selective and non-selective culture media along with an optimized viability real-time PCR utilizing propidium monoazide-quantitative PCR (PMA-qPCR), spheroplast formation and an internal sample process control (ISPC), were comparatively evaluated for monitoring the survival of *C. coli* on fresh lamb meat during refrigeration storage under normal atmospheric conditions. On day zero of three independent experiments, lamb meat pieces were artificially inoculated with *C. coli* and then stored under refrigeration for up to 8 days. Three meat samples were tested on different days and the mean counts were determined per quantification method. An overall reduction of the viable *C. coli* on lamb meat was observed regardless of the applied quantification scheme, but the rate of reduction followed a method-dependent pattern, the highest being observed for colony counting on modified charcoal cefoperazone deoxycholate agar (mCCDA). Univariate ANOVA indicated that the mean counts of viable *C. coli* using PMA-qPCR were significantly higher compared to Columbia blood agar (CBA) plating (0.32 log_10_ cell equivalents, *p* = 0.015) and significantly lower when mCCDA was compared to CBA plating (0.88 log_10_ CFU, *p* < 0.001), indicating that selective culture on mCCDA largely underestimated the number of culturable cells during the course of meat storage. PMA-qPCR outperformed the classical colony counting in terms of quantifying both the culturable and viable but non-culturable (VBNC) *C. coli* cells, which were generated over time on meat and are potentially infectious and equally important from a public health perspective as their culturable counterparts.

## Introduction

*Campylobacter* spp. are ubiquitous in nature and successfully colonize the gastrointestinal tract of warm-blooded animals including food-producing animals (cattle, sheep, pigs and poultry), usually without causing symptoms. Slaughter has been recognized as a crucial stage for fresh meat contamination by thermophilic *Campylobacter* spp. that persists across the meat production chain and represents a public health risk (Lazou et al., 2014a,b; Shange et al., 2019). Campylobacteriosis remains the most commonly reported zoonotic gastrointestinal illness in the European Union (EU) since 2005, commonly related to domestically acquired infection *via* the consumption and handling of raw or undercooked foods of animal origin, particularly poultry meat (EFSA and ECDC, 2019). Human cases are attributed mainly to *Campylobacter jejuni* (*C. jejuni*) followed by *Campylobacter coli*, however, subspecies information is provided only for approximately half of the confirmed cases in the EU since these two subspecies are rarely differentiated in routine diagnostics, although risk factors for human infection may differ between them (Gillespie et al., 2002; Kärenlampi et al., 2007; Roux et al., 2013; EFSA and ECDC, 2019). Nowadays, prevalence, virulence potential and health burden of *C. coli* are regarded substantial and much greater than previously thought (Du et al., 2018; Gomes et al., 2018); it is being increasingly isolated from various samples of animal origin, including meat and offal of small ruminants (Kärenlampi et al., 2007; Løvdal et al., 2011; Voidarou et al., 2011; Nobile et al., 2013; Lazou et al., 2014a,b; Pedonese et al., 2017; Di Giannatale et al., 2019), whereas, a tendency towards increased antimicrobial resistance of its isolates has been recently observed compared to *C. jejuni* (Voidarou et al., 2011; Du et al., 2018; García-Sánchez et al., 2018; Di Giannatale et al., 2019). Data regarding the survival of *C. coli* cells on fresh meat, other than poultry, during refrigeration in the domestic environment are scarce, although cross-contamination incidents are likely to occur, since consumer awareness regarding the risk of food contamination by *Campylobacter* in the household remains deficient (Hansson et al., 2018).

A laborious and time-consuming procedure lasting several days is required for the *in vitro* detection, enumeration and biochemical confirmation of *Campylobacter* colony-forming units (CFU) according to the ISO 10272-2 standard colony-count technique, which is the only universally accepted, adequately validated, and standardized method (Elizaquível et al., 2014; Zeng et al., 2016; ISO 10272-2:2017, 2017). Another major drawback for the routine implementation and universal acceptance of classical plate counting is the inability to detect viable but non-culturable (VBNC) cells. The VBNC state is regarded as an adaptive response of bacterial cells to stressful environmental conditions, which render them unable to proliferate on typical culture media, including enrichment media designed to resuscitate injured cells (Baffone et al., 2006; Oliver, 2010; Oh et al., 2015). Campylobacters have been reported to enter the VBNC state when exposed to low temperatures and atmospheric oxygen (e.g., chilling chambers); however, VBNC bacteria remain capable of switching to the infectious state once inside the human host (Oliver, 2010; Chaisowwong et al., 2012; Stingl et al., 2012; Li et al., 2014; Oh et al., 2015). Although the reliable detection of both culturable and VBNC cells is equally important in view of public health inferences, culturable *Campylobacter* bacteria in any given sample can be detected and quantified by traditional plate counting in contrast to their VBNC counterparts that will remain undetected (Chaisowwong et al., 2012; Elizaquível et al., 2014; Krüger et al., 2014; Zeng et al., 2016). Nevertheless, the rapid and reliable detection and quantification of viable *Campylobacter* directly in samples complies with the modern requirements of research and food industry, especially regarding products with limited shelf life, such as fresh meat, but the utilization of culture-based methods is hampered by the aforementioned shortcomings (Elizaquível et al., 2014; Krüger et al., 2014; Duarte et al., 2015; Papić et al., 2017).

Real-time PCR (quantitative PCR; qPCR) could serve as an alternative method for the rapid, high throughput, and specific detection of foodborne pathogens in general and *Campylobacter* in particular in food samples, but its inability to differentiate between the amplified DNA of viable and dead cells results in indefinite information regarding the pertained public health risk (Fittipaldi et al., 2012; Elizaquível et al., 2014; Zeng et al., 2016). In order to overcome this impediment, viability PCR (v-PCR) utilizing qPCR and nucleic acid intercalating dyes has been developed to provide rapid quantification combined with viability information (Nocker et al., 2006). This technique is based on the viability criterion of cell membrane integrity, which operates as a physical barrier against the entrance of DNA-intercalating dyes, principally propidium monoazide (PMA), into live bacterial cells (both culturable and nonculturable). The theoretical mode of action includes the selective entrance of PMA into dead bacterial cells *via* their compromised-membranes and the DNA cleavage upon photoactivation that prohibits its subsequent amplification during qPCR (Nocker et al., 2006; Wagner et al., 2008; Varma et al., 2009). However, applications of v-qPCR protocols with PMA (PMA-qPCR) on meat samples have encountered various challenges and limitations attributed to a rather conditional and not absolute suppression of qPCR signals originating from dead *Campylobacter* bacteria (false-positive signals) due to a complex set of parameters including experimental, target and sample features (Nocker et al., 2006; Pacholewicz et al., 2013; Krüger et al., 2014; Duarte et al., 2015; Vondrakova et al., 2018). The development of a PMA-qPCR assay utilizing spheroplast formation as a pretreatment for enhancing the selective entrance of PMA to dead cells and an internal sample process control (ISPC), unique for any given sample, that could address false-positive signals originating from dead cells, has only recently been achieved using *C. coli* as a bacterial model (Lazou et al., 2019).

The aim of the present study was to address method-dependent implications during the quantification of viable *C. coli* cells on meat over time. The aforementioned PMA-qPCR utilizing spheroplast formation and ISPC, along with traditional colony counting on selective and non-selective solid media were comparatively evaluated for monitoring the survival of *C. coli* on fresh lamb meat during refrigeration storage under normal atmospheric conditions.

## Materials and Methods

### General Experimental Scheme

**Figure 1:**
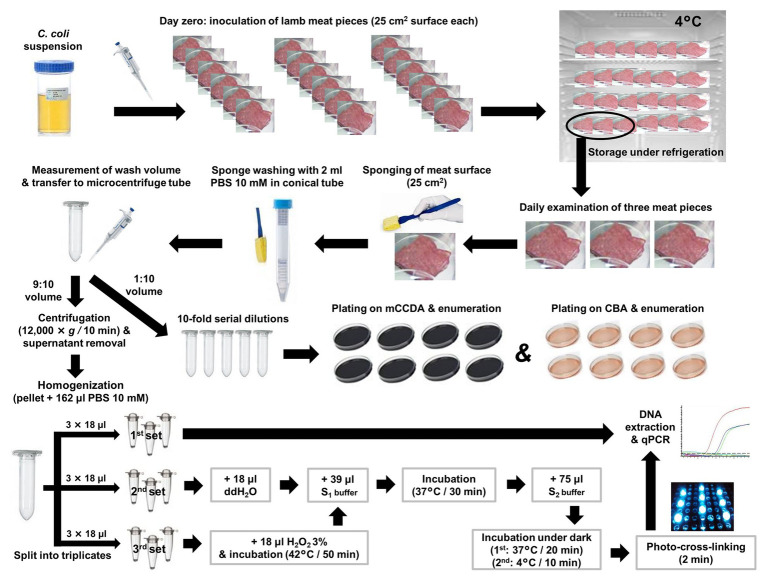
General experimental scheme for the quantification of culturable, total, and viable *C. coli* cells on artificially inoculated lamb meat pieces using plate counting, qPCR (control) and PMA-qPCR, respectively. S_1_ buffer: 10 mM Phosphate Buffer Saline (PBS; pH 7.0); 0.5 M Sucrose; 1.0 mg/ml Lysozyme; 50 mM EDTA. S_2_ buffer: 10 mM PBS (pH 7.0); 50 mM EDTA; 100 μM PMA. ddH_2_O: double-distilled water.

Three independent experiments were performed at different time frames, following the general experimental scheme as illustrated in Figure 1. On day 0 of each experiment, individual lamb meat pieces were inoculated with a defined *C. coli* population and then placed in a refrigerator set at 4°C. Temperature was recorded daily and three individual meat pieces were examined at each time-point, in order to quantify the average viable *C. coli* count by plating on two culture media and by using PMA-qPCR. In the first experiment, the average viable *C. coli* count of three samples was quantified daily for a week (in total, 21 meat pieces), at the end of which meat spoilage was visually evident. Based on the results of the first experiment, viable *C. coli* counts were quantified every other day from day 0 to 8 during the second and third experiment (in total, 15 meat pieces per experiment).

### Preparation of *Campylobacter coli* Suspension

On each experimental occasion, *C. coli* ATCC® 43478™ (LGC Standards GmbH, Wesel, Germany), previously stored at −80°C in nutrient broth No. 2 (Oxoid, Basingstoke, England) supplemented with 5% lysed horse blood (Oxoid) and 20% glycerol (BDH VWR, Lutterworth, England), was recovered on Columbia blood agar (CBA; bioMérieux, Marcy-l’Étoile, France), then isolated on modified charcoal cefoperazone deoxycholate agar (CCDA; Oxoid) and subcultured once more on CBA. Plate incubation was always performed at 41.5 ± 1°C for 24 h under microaerobic conditions (GENbox microaer and GENbox jar, bioMérieux). For the generation of pure *C. coli* suspensions, one pure colony from CBA was inoculated in 100 ml of cation-adjusted Mueller-Hinton broth (CAMHB; BBL™ BD, Franklin Lakes, NJ) following incubation at 37 ± 1°C for 20 ± 2 h under microaerobic conditions. The growth state of the bacterial culture was approximately estimated at the end of the incubation by measuring the optical density (OD) at 600 nm using a spectrophotometer (BioPhotometer®; Eppendorf, Hamburg, Germany). The estimated count was confirmed by subjecting three aliquots of 100 ml to qPCR, as described below, and by performing 10-fold serial dilutions in buffered peptone water (BPW, LabM, United Kingdom) followed by spread plating on double CBA plates.

### Artificial Inoculation of Lamb Meat and Sampling

For each experiment, a deboned lamb leg was purchased from the retail market and tested for the presence of *C. coli* as follows: a representative sample of 25 g of meat was homogenized in 225 ml of BPW; subsequently, five aliquots of 1 ml each were subjected to DNA extraction and qPCR. Once the *C. coli*-free status of the lamb meat was confirmed, meat pieces (25 cm^2^ total surface) were generated (*n* = 21 in the first experiment; *n* = 15 in the second and third experiments) by using sterile lancet and forceps and placed individually in sterile petri dishes, without applying any method of decontamination of the autochthonous microbiota. The samples for each experiment were inoculated on day 0 by uniformly applying 100 μl of a pure *C. coli* suspension in CAMHB (as described previously) on their surface, and then stored at 4°C. The first three meat samples were tested following 4 ± 1 h of storage at 4°C (allowed time for adherence of the bacterial cells to the meat surface). A sterile cellulose sponge (Whirl-Pak® Speci-Sponge®, Nasco, NY, United States), cut aseptically (0.5 cm × 1.0 cm) and pre-moistened with phosphate-buffered saline (PBS) 10 mM (Sigma-Aldrich, Steinheim, Germany), was used to sample each meat surface (Figure 1) by 10 horizontal, 10 vertical, and 10 diagonal swabbings. The sponge was then transferred in a conical centrifuge tube (15 ml, Falcon®, NY, United States) containing 2 ml of PBS 10 mM and homogenized for 60 s, using a vortex shaker (IKA®-Werke GmbH & Co. KG, Staufen, Germany). Subsequently, the sponge was drained by squeezing it against the tube wall and discarded. The volume of the remaining wash suspension was determined by a calibrated adjustable micropipette (Eppendorf Research® plus G, variable 100–1000 μl, NY, United States) while being transferred to a microcentrifuge tube (2.0 ml, Kisker Biotech GmbH & Co, Steinfurt, Germany). One tenth of the wash suspension was used for 10-fold serial dilutions and culture-based enumeration, whereas the remaining quantity (nine parts out of 10) was subjected to qPCR-based quantification (Figure 1).

### DNA Extraction and qPCR Quantification

After centrifugation of the 9:10 volume of the wash suspension (12,000 × *g* for 10 min), the supernatant containing any extracellular DNA was discarded, and the pelleted bacterial cells were obtained in 162 μl PBS 10 mM. This suspension was then homogenized and nine aliquots (18 μl each) were generated and transferred to equal number of transparent PCR tubes (0.2 ml; Kisker Biotech GmbH & Co). The treatments of an optimized PMA-qPCR utilizing spheroplast formation (induced by lysozyme and EDTA) and ISPC, as described by Lazou et al. (2019), were applied. In brief, the first set of thee aliquots was directly subjected to DNA extraction and qPCR in order to quantify the total *C. coli* cell equivalents (quantification controls). The second set was subjected to spheroplast formation and PMA treatment, and the third set underwent inactivation by 1.5% hydrogen peroxide (H_2_O_2_) at 42°C for 50 min, followed by spheroplast formation and PMA treatment. The final concentration of EDTA in each aliquot subjected to spheroplasting was 25 mM. All aliquots were finally subjected to DNA extraction and qPCR (Figure 1). A protocol previously described and evaluated for *Campylobacter* (Lazou et al., 2014b), was used for the extraction of genomic DNA. In brief, each aliquot was mixed with 100 μl of “Lysis buffer I” (50 mM Tris-HCl, 50 mM EDTA, 4 M Guanidinium hydrochloride-GuHCl, 10 mM CaCl2, 1% Triton X-100, and 2% *N*-lauroyl-sarcosine, pH 7.5) and 25 μl of proteinase K (0.56 mg; New England Biolabs, Ipswich, MA, United States), and mixtures were incubated at 56°C for 1 h. Following this step, 250 μl of “Lysis buffer II” (50 mM Tris-HCl, 25 mM EDTA, 8 M GuHCl, 3% Triton X-100, and 3% N-lauroyl-sarcosine, pH 6.3) were added and mixtures were incubated at 70°C for 10 min. Absolute ethanol (250 μl) was added to the lysates, and each mixture was passed through a silica column (FT-2.0; Kisker Biotech GmbH & Co) by centrifugation (8,000 × *g*). Columns were washed three times, once with “Wash buffer I” (25 mM Tris-HCl, 4 M GuHCl, and 50% ethanol, pH 6.6), and twice with “Wash buffer II” (10 mM Tris-HCl, 0.1 M NaCl, and 80% ethanol, pH 6.6), followed by elution in 80 μl TE buffer.

The qPCR assay, as described by Lazou et al. (2019), targeting the serine hydroxymethyltransferase (*gly*A) single-copy gene (one genome copy represents one cell equivalent) was applied. Briefly, the primers used were Cc-F (5'-TGTAAAACCAAAGCTTATCGTGTGC-3') and Cc-R (5'-AGTCCAGCAATGTGTGCAATG-3'), along with the Cc-FAM TaqMan probe (5'-6-FAM-AGCTCCAACTTCATCCGCAATCTCTCT-BHQ1-3'). The 50 μl qPCR reaction was comprised by 1× ThermoPol® DF reaction buffer (New England Biolabs), 0.2 μM of each primer, 0.4 μM of the probe, 0.2 mM of each dNTP, 2.5 mM MgSO_4_, 4 U HotStarTaq DNA polymerase (Qiagen, Hilden, Germany), and 15 μl of DNA extract. Cycling conditions were as follows: 95°C for 15 min, followed by 45 cycles in two steps: (i) 95°C for 30 s and (ii) 60°C for 50 s. Fluorescence was measured at the end of each cycle. The qPCR assay exhibited a linear range of 10^0^–10^7^ copies per reaction (*R*^2^ > 0.99), with a PCR efficiency of 93.4% and an LOD of 7.046 copies per reaction (95% CI). The standard curve was described by the equation y = 10^[(41.763 ‐ Ct)/3.491)]^, where y stands for the genome copies per qPCR reaction (Lazou et al., 2019).

Each obtained C*t* value during qPCR was transformed to cell equivalents based on the standard curve and the corresponding dilution factors with regard to the original sample. In particular, the *C. coli* count (*y*) per meat surface (25 cm^2^) was calculated by the following formula, taking into account the previously described standard curve equation (Lazou et al., 2019):

*y* = (10 ^[(41.763-*Ct*)/3.491]^ × *a* × *b* × *c*) /*d*

where,

*Ct* = the Cycle threshold.
*a* = the ratio DNA_total_/DNA_assay,_ with DNA_total_ accounting for the total extracted pure DNA volume (80 μl) and DNA_assay_ for the volume of the DNA template (15 μl) used per qPCR assay (80 μl/15 μl = 5.33).
*b* = the total number of aliquots of the bacterial pellet homogenate that were subjected to qPCR (b = 9).
*c* = the reduction coefficient of the *C. coli* cells that were present in the 9:10 volume of the wash suspension with respect to the original wash suspension (*c* = 1.11).
*d* = the recovery (%) of *C. coli* from the meat surface during swabbing (mean value of the total genome copies of the quantification controls divided by the mean value of the total genome copies of the original *C. coli* suspension inoculated on meat, as obtained by qPCR).

The three individual cell equivalent counts corresponding to the three meat pieces examined per day were used for the calculation of the average cell equivalent count on lamb meat for that particular day in each experiment. The mean count of the second set of triplicate aliquots (PMA-treated spheroplasts) minus the mean count of the third set of triplicate aliquots (ISPC) was considered as the viable cell equivalent count obtained by PMA-qPCR for each meat sample. The artificial inoculation of lamb meat pieces with a qPCR-defined *C. coli* population (quantified as cell equivalents) and the inclusion of quantification controls enabled a direct calculation of the recovery (%) of campylobacters by the applied non-destructive method of swabbing.

### Culture-Based Quantification

For the culture-based quantification, one tenth of the washed suspension was subjected to 10-fold serial dilutions in BPW (Merck) and 100 μl of each dilution were inoculated on double plates both of mCCDA, which is the solid selective medium of first choice according to the standard method for the enumeration of *Campylobacter* spp. (ISO 10272-2:2017, 2017), and of the non-selective CBA (no CBA plates were used in the first experiment) followed by incubation at 41.5 ± 1°C for 24 to 48 h under microaerobic conditions (Figure 1). The plates were initially checked after the first 24 h of incubation to assess the extent of colony growth on the agar surface and to enumerate the presumptive *Campylobacter* colonies at this stage, followed by incubation under microaerobic conditions for another day and final enumeration at 48 h. The aforementioned qPCR assay was applied for the confirmation of 10 presumptive *Campylobacter* colonies on each selected plate and the weighted mean was used for the calculation of CFU (Jarvis, 2016). For the quantification of the viable *C. coli* on each meat surface (CFU/25 cm^2^), the reduction coefficient (*c* = 10) and the recovery (*d*) were considered, as described previously. The three obtained CFU counts corresponding to the three meat pieces examined per day were used for the calculation of the average CFU count on lamb meat for each particular day in each experiment.

### Statistical Analysis

All counts of viable *C. coli* on lamb meat, as calculated per day by each applied method, were log_10_-transformed to be further analyzed. Microsoft Office Excel 2010 (Microsoft Corporation, Redmond, Washington, United States) was used for the generation of linear regression equations, which were subsequently used for the calculation of the average log-rank reduction of viable campylobacters per quantification method in each experiment.

Univariate ANOVA (SPSS v21, IBM) were used to assess the effect of quantification method (3 levels) on the log-transformed counts of viable *C. coli* in lamb meat, adjusting for the fixed effects of experimentation number (3 levels) and testing day (4 levels) as presented below:

Cb_abc_ = m_abc_ + M_a_ + E_b_ + D_c_ + e_abc_

where,

Cb_abc_ = Log-transformed value of viable *C. coli* population per 25 cm^2^ of lamb meat surface.
m_abc_ = overall mean.
M_a_ = Quantification method of viable *C. coli* (a = 3 levels: 1 = PMA-qPCR, 2 = mCCDA, and 3 = CBA).
E_b_ = Experiment number (b = 3 levels: 1st, 2nd and 3rd experiment).
D_c_ = Testing day (c = 4 levels: day 0, 2, 4, and 6).
e_abc_ = SE.

Statistical significance was set at 0.05 level.

## Results

**Figure 2:**
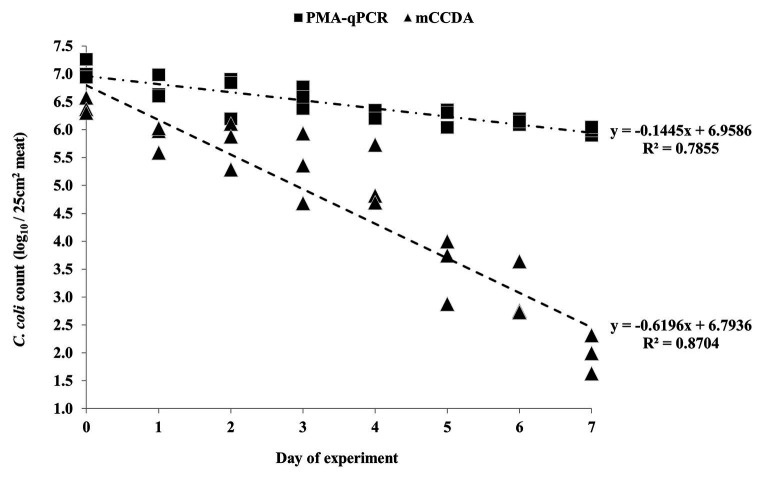
Quantification of *C. coli* on three lamb meat pieces per day during the first independent experiment, as obtained by PMA-qPCR (log_10_ cell equivalents/25 cm^2^ meat surface) and plating on mCCDA (log_10_ CFU/25 cm^2^ meat surface). Each dot represents one lamb meat sample. The linear regression equations and *R*^2^ values are shown in the diagram.

**Figure 3:**
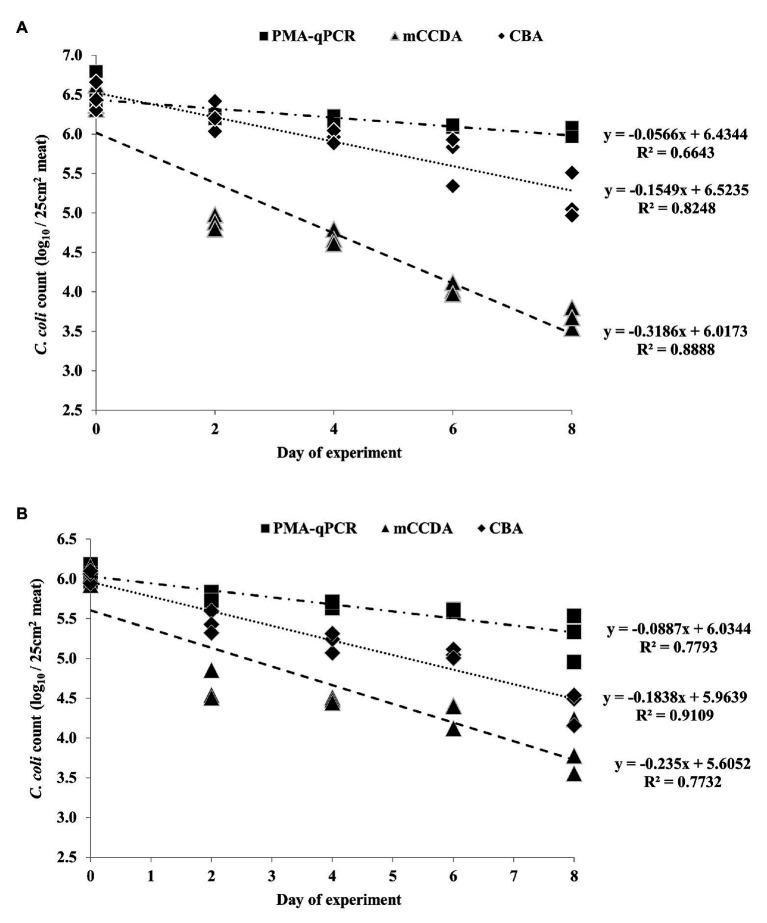
Quantification of *C. coli* on three lamb meat pieces per day during the second **(A)** and third **(B)** independent experiments, as obtained by PMA-qPCR (log_10_ cell equivalents/25 cm^2^ meat surface), and plating on mCCDA (log_10_ CFU/25 cm^2^ meat surface) and CBA (log_10_ CFU/25 cm^2^ meat surface). Each dot represents one lamb meat sample. The corresponding linear regression equations and *R*^2^ values are shown in the diagram.

All the applied quantification methods indicated an overall reduction of viable *C. coli* bacteria under refrigeration storage of lamb meat (Figures 2, 3). Although the initially inoculated *C. coli* population on the lamb meat pieces was random in each experiment, the difference of the average viable counts among the quantification methods on day 0 was not statistically significant (*p* > 0.05) in all experiments (Table 1). However, the observed degree of reduction of the viable campylobacters over time was method-dependent; the most dramatic being observed for colony counting on mCCDA and the least for PMA-qPCR. In particular, the average count (three lamb meat samples) of viable *C. coli* obtained by PMA-qPCR were 1.42–3.99 log_10_ higher than those obtained by mCCDA plating on the last day of monitoring as regards the three independent experiments. Interestingly, the difference in the culturable *C. coli* cells between the two solid media peaked over time to result on the last day in CBA yielding higher average counts by 0.54–1.51 log_10_ compared to mCCDA among the three experiments (Table 1).

**Table 1:** Average daily count (±SD) of viable *Campylobacter coli* cells per day of experiment on lamb meat (surface 25 cm^2^) as obtained by propidium monoazide-quantitative PCR (PMA-qPCR; log_10_ cell equivalents), Columbia blood agar (CBA) plating (log_10_ CFU) and modified charcoal cefoperazone deoxycholate agar (mCCDA) plating (log_10_ CFU).

	1st Experiment		2nd Experiment			3rd Experiment		
DAY	Quantification method		Quantification method			Quantification method		
	PMA-qPCR	mCCDA	PMA-qPCR	mCCDA	CBA	PMA-qPCR	mCCDA	CBA
0	7.06 ± 0.17	6.42 ± 0.14	6.52 ± 0.24	6.43 ± 0.16	6.47 ± 0.18	6.07 ± 0.11	6.05 ± 0.12	6.04 ± 0.08
1	6.73 ± 0.21	5.86 ± 0.24						
2	6.64 ± 0.39	5.76 ± 0.42	6.22 ± 0.03	4.88 ± 0.09	6.22 ± 0.19	5.79 ± 0.06	4.63 ± 0.19	5.45 ± 0.14
3	6.57 ± 0.19	5.32 ± 0.63						
4	6.27 ± 0.07	5.08 ± 0.56	6.19 ± 0.06	4.69 ± 0.09	5.96 ± 0.08	5.66 ± 0.04	4.48 ± 0.03	5.21 ± 0.12
5	6.23 ± 0.17	3.54 ± 0.59						
6	6.14 ± 0.05	3.04 ± 0.52	6.10 ± 0.02	4.04 ± 0.07	5.70 ± 0.32	5.61 ± 0.02	4.31 ± 0.16	5.05 ± 0.06
7	5.97 ± 0.08	1.98 ± 0.34						
8			6.01 ± 0.06	3.67 ± 0.13	5.17 ± 0.29	5.28 ± 0.29	3.86 ± 0.35	4.40 ± 0.21

Based on the generated linear regression equations (Figures 2, 3), the average daily log-rank reduction rate (mean ± SD) of viable *C. coli* on lamb meat in all three experiments was 0.10 ± 0.044, 0.17 ± 0.020, and 0.39 ± 0.202 log_10_ cell equivalents or CFU regarding PMA-qPCR, plating on CBA and mCCDA, respectively. Likewise, the calculated overall log-rank reduction (mean ± SD) of viable *C. coli* on lamb meat in all three experiments was 0.77 ± 0.36, 1.36 ± 0.16, and 3.21 ± 1.58 log_10_ cell equivalents or CFU for PMA-qPCR, and plating on CBA and mCCDA, respectively.

**Figure 4:**
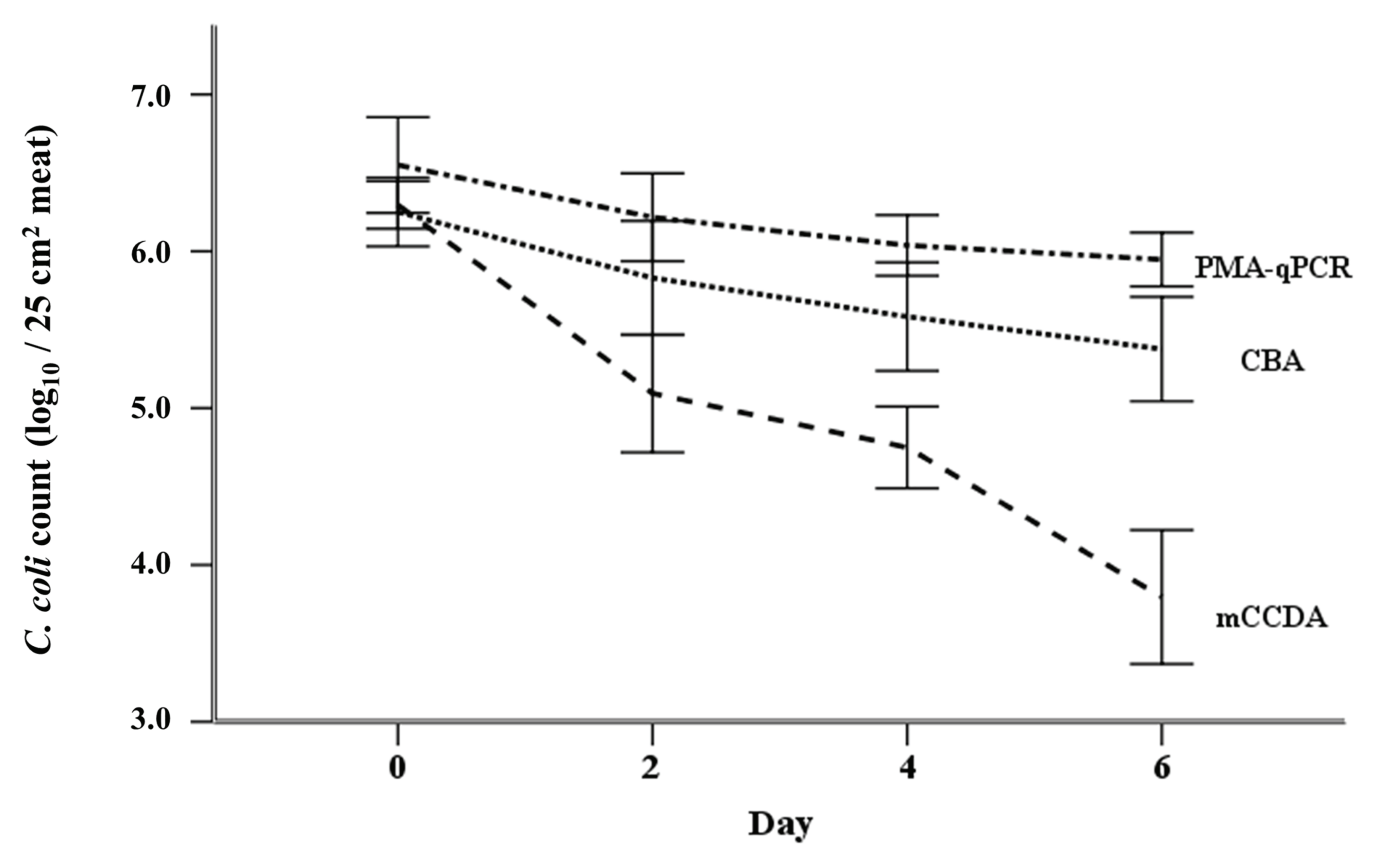
Regression model-predicted curves of the viable *C. coli* counts (mean ± SD) per applied quantification method (PMA-qPCR, CBA, and mCCDA plating) as regards the first 6 days after the artificial inoculation (day 0) of lamb meat samples.

The applied ANOVA accounted for 72.3% of the total variance of the log-rank counts of *C. coli*, and was found to predict the dependent variable significantly well (*p* < 0.001). The overall effects of quantification method, experiment number, and test day were significant at *p* < 0.001 level. In general, in all three experiments, the obtained counts of viable *C. coli* cells using PMA-qPCR were significantly higher compared to CBA plating (*ca*. 0.32 log_10_ cell equivalents, *p* = 0.015). Similarly, CBA plating counts were significantly higher compared to mCCDA plating (*ca*. 0.88 log_10_ CFU, *p* < 0.001). The statistical model predicted decreasing counts of viable *C. coli* cells from day 0 to day 6 (Figure 4). Specifically, viable *C. coli* counts were significantly higher on days 0 (ca. 1.38 log_10_ cell equivalents, *p* < 0.001), 2 (ca. 0.70 log_10_ cell equivalents, *p* < 0.001), and 4 (ca. 0.44 log_10_ cell equivalents, *p* = 0.001) compared to day 6.

**Figure 5:**
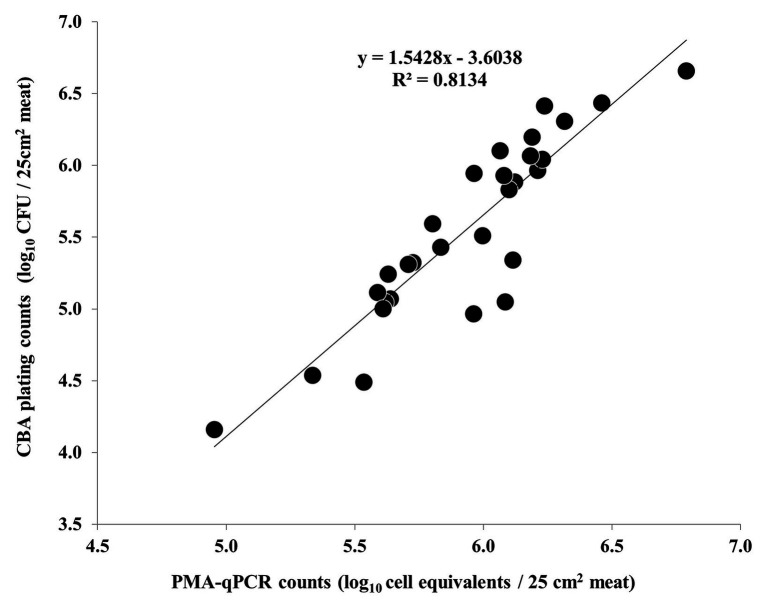
Correlation of *C. coli* log_10_ cell equivalent counts obtained by PMA-qPCR and log_10_ CFU counts obtained by plating on CBA during the second and third independent experiments (each dot represents an individual meat sample). The linear regression equation and *R*^2^ value are shown in the diagram.

**Figure 6:**
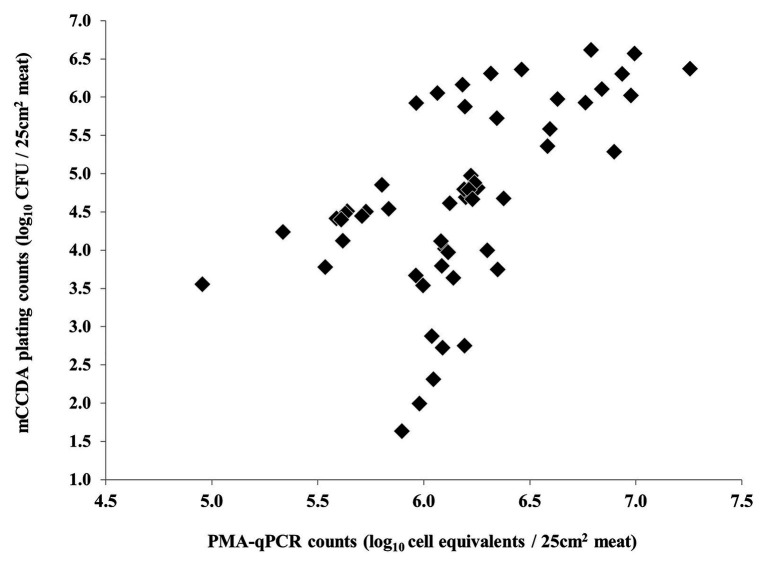
Non-linear correlation of *C. coli* log_10_ cell equivalent counts obtained by PMA-qPCR and log_10_ CFU counts obtained by plating on mCCDA during all three independent experiments (each dot represents an individual meat sample).

Nonetheless, a strong correlation between the results of PMA-qPCR and plating on CBA was observed regarding the second and third experiments (Figure 5). On the contrary, the counts obtained by PMA-qPCR and plating on mCCDA were not strongly correlated at any time-point during the study (Figure 6).

## Discussion

The majority of available studies on the detection and/or quantification of *Campylobacter* on meat are relevant to poultry meat and products thereof. To the best of our knowledge, this is the first study regarding the survival of *C. coli* on chilled lamb meat in general and the parallel quantification of its viable cells by CFU determination and viability PMA-qPCR quantification in particular.

An overall decline of viable *C. coli* bacteria has been observed in the current study irrespectively of the applied quantification method. The reduction of viable *Campylobacter* counts during refrigeration of fresh meat is rather expected, since campylobacters are regarded unable to multiply in temperatures lower than 30°C despite their prolonged survival at 4°C (Jacobs-Reitsma et al., 2008). Data on the survival of *C. coli* on poultry meat in the concurrent presence of native microbiota have been reported by Oyarzabal et al. (2010) who applied direct plating on mCCDA. In particular, the log-rank decline of *C. coli* (0.83 ± 0.41 log_10_ CFU/g) was found to be lower than that of *C. jejuni* (2.01 ± 0.48 log_10_ CFU/g) on artificially inoculated chicken meat maintained at 4°C for 14 days. Differences in the survival potential of *Campylobacter* on poultry meat and corresponding preparations have been observed between different subspecies, as well as between different strains within the same species. The population of two distinct *C. coli* strains on the skin of fresh poultry meat has been observed to drop by 2.6 ± 0.43 and 3.4 ± 0.07 log_10_ CFU/cm^2^ on mCCDA plates over a period of 9 days at 4°C (El-Shibiny et al., 2009). The importance of the food matrix for the survival of campylobacters has been substantiated in previous studies. In particular, differences have been observed in the decline rate of viable campylobacters between different samples originating from the same carcass, for example fresh poultry meat and corresponding skin (Oyarzabal et al., 2010). Meat surface microbiota is thought to reduce the relative pressure of oxygen due to respiration, thereby reducing the oxidative stress level for campylobacters and promoting their survival (El-Shibiny et al., 2009). Moreover, *Campylobacter* strains appear to exhibit different survival characteristics when studied individually though *C. coli* has emerged as more aerotolerant than *C. jejuni* (Karki et al., 2018). Important differences between strains as regards residual viable cells have also been reported in biofilms of *Listeria monocytogenes* cultivated on stainless steel as detected by PMA-qPCR and epifluorescence microscopy indicating a diverse potential of adaptation and survival within the same species (Brauge et al., 2019). The fact that only one strain of *C. coli* was used in the current study is a recognized limitation regarding the overall survival potential of this species on lamb meat and, therefore, more studies utilizing various strains of this pathogen are needed to provide better insight in this context.

Apart from the overall reduction of the viable *C. coli* on lamb meat under refrigeration regardless of the applied quantification scheme, the relative degree of reduction in the current study followed a method-dependent pattern. Although on day zero all methods yielded approximately similar counts of viable *C. coli* (*p* > 0.05), an ascending order in the reduction rate was observed in the following days regarding PMA-qPCR, CBA plating and mCCDA plating, respectively. Accordingly, at the end of the monitoring period in all experiments, significantly higher counts (*p* < 0.05) of viable *C. coli* cells were obtained by PMA-qPCR compared to CBA plating and by CBA compared to mCCDA plating. These findings can be further interpreted in view of the quantification capacities of each method. Selective factors present in mCCDA, such as antibiotics, have been reported to impose an additional hurdle to already stressed bacteria, thus, reducing the observed CFU counts when compared to non-selective media, such as CBA (Bell et al., 2005). Lower CFU counts of *Campylobacter* on poultry meat have also been observed on mCCDA plates in comparison to other selective media (Habib et al., 2011). Likewise, in the current study, the observed CFU count of *C. coli* on lamb meat was higher on CBA plates compared to mCCDA plates on every occasion. However, the *C. coli* cells that were conceivably transformed to VBNC due to the exposure to the microenvironment of meat, particularly the stressful atmospheric oxygen and refrigeration temperature, were, by definition, unable to form visible colonies and be enumerated on mCCDA and CBA plates. VBNC cell population can be estimated as the difference between culturable cells on solid media and viable cells as determined by PMA-qPCR (Lazou et al., 2019; Lv et al., 2020). Therefore, VBNC bacteria on lamb meat were quantified as viable along with the culturable ones by the PMA-qPCR assay, which resulted in higher counts compared to plating on CBA for each day of quantification during the present study. Higher counts of viable *Campylobacter* obtained by PMA-qPCR compared to culture on mCCDA plates have also been reported in other studies regarding meat samples (Pacholewicz et al., 2013; Duarte et al., 2015). As regards bias, any potential overestimation of viable *C. coli* cells due to an insufficient reduction of the signal from dead cells (false positive signals) has been successfully minimized in the present study by the use of the ISPC comprised of inactivated cells subjected to the same treatments and with an equal population as the ones used for enumeration. The importance of developing and utilizing ISPC during the application of PMA-qPCR assays in order to improve cultivation-independent quantification of thermotolerant *Campylobacter* has been emphasized in previous studies (Lazou et al., 2019; Pacholewicz et al., 2019). A former application of the same PMA-qPCR protocol used in the present study to meat samples artificially inoculated with *C. coli* revealed no statistical difference between the viable-cell counts obtained by PMA-qPCR and plating on mCCDA but only regarding bacteria in the exponential phase of growth (Lazou et al., 2019).

The unsuitability of CFU count as a reliable parameter for reproducible quantification and assessment of the risk for infection by *Campylobacter* detected on food products has been previously reported by Krüger et al. (2014). More specifically, a large variability (>2.0 log_10_) of the CFU of *Campylobacter* that managed to grow on CBA plates has been observed between the exponential and stationary phase of growth of a single strain. In addition, the comparative enumeration of CFU, the microscopically determined cell counts, and DNA content of *C. jejuni* and *C. coli* indicated that colonies were formed by 25.0, 10.0, and 0.3% of bacterial cells at the early stage of the exponential phase, the late stage of the exponential phase, and the stationary phase, respectively. Consequently, classical culture cannot provide quantification data on the total *Campylobacter* population that originally contaminated a food item during its production and underestimates the potentially infectious population of the pathogen, which includes any VBNC subpopulation present. This fact is further reinforced by the findings of the present study; the CFU counts obtained on mCCDA, which is the solid selective medium for the standard colony-count technique for *Campylobacter* (ISO 10272-2:2017, 2017), indicated a more dramatic reduction and significantly (*p* < 0.001) underestimated the viable *C. coli* compared to the PMA-qPCR method during the storage of lamb meat at 4°C. The application of PMA-qPCR has also highlighted that *Campylobacter* survival in raw milk can be largely underestimated when relying merely on CFU data (Wulsten et al., 2020). Accordingly, PMA-qPCR has been reported to detect and quantify VBNC bacteria in 90.48% of culture-negative fresh and processed meat samples regarding *Staphylococcus aureus*, *Bacillus cereus*, *Clostridium perfringens*, and Enterobacteriaceae (Abd El-Aziz et al., 2018).

For meat and products thereof, the original contamination with *Campylobacter* occurs during slaughter whereas the consumer’s exposure to the pathogen takes place at the time of meat preparation and consumption several days later. Important knowledge gaps regarding prevention, control and diagnosis of campylobacteriosis have been recently highlighted by Hansson et al. (2018). Consumer education on domestic hygiene in order to prevent transfer of *Campylobacter* from raw to ready-to-eat foods has been identified as an emerging gap for the public health domain. Indeed, relevant epidemiological data reveal that a significant portion of the reported cases of foodborne illness is attributed to poor practices of the final consumer regarding handling of contaminated foods in the household (Redmond and Griffith, 2003). The results of the present study indicate the survival and subsequent diminutive reduction of viable *C. coli* cells on lamb meat under refrigeration storage, which is in accordance with previously reported findings for other types of meat (Jacobs-Reitsma et al., 2008; El-Shibiny et al., 2009; Oyarzabal et al., 2010). The rather slight reduction of the viable *C. coli* population on lamb meat during storage for several days and until its spoilage, highlights that cross-contamination by infectious campylobacters is likely to occur within the household environment. Therefore, the impact of the applied quantification method on the obtained viable *Campylobacter* counts entails considerable public health implications especially in view of the multifactorial induction of the VBNC state and the source attribution of campylobacteriosis. The longer the period between the point of food contamination and the time of sampling, the larger the VBNC bacterial population present and the corresponding deviation of the CFU estimate from the potentially infectious *Campylobacter* cells, particularly if the initial contamination is rather low.

## Conclusion

In conclusion, the optimized PMA-qPCR outperformed traditional colony counting as regards the monitoring of viable *C. coli* on lamb meat during refrigeration storage under normal atmospheric conditions. Utilization only of classical plating for the detection and enumeration of viable campylobacters on meat overlooks by definition the presence of any VBNC counterparts, which are potentially infectious and equally important from a public health perspective. Moreover, standard colony counting on selective solid media, such as mCCDA, underestimates even the culturable *Campylobacter* cells in comparison to non-selective ones, such as CBA. In contrast, viability PMA-qPCR can be a useful tool for the concurrent quantification of both culturable and VBNC campylobacters on meat. Therefore, optimization of molecular techniques, ‘such as PMA-qPCR,’ in order to achieve reliable *Campylobacter* quantification would considerably reduce the requirements in terms of time and cost. Such efforts would further facilitate a broader *Campylobacter* risk assessment and successful surveillance throughout all stages in the food supply chain.

## Data Availability Statement

The original contributions presented in the study are included in the article/supplementary material. Further inquiries can be directed to the corresponding authors.

## Author Contributions

TL, EI, and CD: design of the study. TL and SC: preparation of laboratory media. TL: experiment performance. TL, AG, and CD: data analysis. TL, AG, SC, and CD: manuscript preparation. All authors contributed to the article and approved the submitted version.

### Conflict of Interest

The authors declare that the research was conducted in the absence of any commercial or financial relationships that could be construed as a potential conflict of interest.
